# Combined Proteome and Transcriptome Analysis of Heat-Primed Azalea Reveals New Insights Into Plant Heat Acclimation Memory

**DOI:** 10.3389/fpls.2020.01278

**Published:** 2020-08-19

**Authors:** Xiuyun Wang, Zheng Li, Bing Liu, Hong Zhou, Mohamed S. Elmongy, Yiping Xia

**Affiliations:** ^1^ Genomics and Genetic Engineering Laboratory of Ornamental Plants, Department of Horticulture, College of Agriculture and Biotechnology, Zhejiang University, Hangzhou, China; ^2^ Department of Vegetable and Floriculture, Faculty of Agriculture, Mansoura University, Mansoura, Egypt

**Keywords:** heat acclimation, acquired thermotolerance, photosynthesis, Rubisco activase, heat shock protein, proteome, transcriptome, *Rhododendron hainanense*

## Abstract

Plants can obtain superinduction of defense against unpredictable challenges based on prior acclimation, but the mechanisms involved in the acclimation memory are little known. The objective of this study was to characterize mechanisms of heat acclimation memory in *Rhododendron hainanense*, a thermotolerant wild species of azalea. Pretreatment of a 2-d recovery (25/18°C, day/night) after heat acclimation (37°C, 1 h) (AR-pt) did not weaken but enhanced acquired thermotolerance in *R. hainanense* with less damaged phenotype, net photosynthetic rate, and membrane stability than non-acclimation pretreated (NA-pt) plants. Combined transcriptome and proteome analysis revealed that a lot of heat-responsive genes still maintained high protein abundance rather than transcript level after the 2-d recovery. Photosynthesis-related genes were highly enriched and most decreased under heat stress (HS: 42°C, 1 h) with a less degree in AR-pt plants compared to NA-pt. Sustainably accumulated chloroplast-localized heat shock proteins (HSPs), Rubisco activase 1 (RCA1), beta-subunit of chaperonin-60 (CPN60β), and plastid transcriptionally active chromosome 5 (pTAC5) in the recovery period probably provided equipped protection of AR-pt plants against the subsequent HS, with less damaged photochemical efficiency and chloroplast structure. In addition, significant higher levels of RCA1 transcripts in AR-pt compared to NA-pt plants in early stage of HS showed a more important role of RCA1 than other chaperonins in heat acclimation memory. The novel heat-induced RCA1, rather than constitutively expressed RCA2 and RCA3, showed excellent thermostability after long-term HS (LHS: 42/35°C, 7 d) and maintained balanced Rubisco activation state in photosynthetic acclimation. This study provides new insights into plant heat acclimation memory and indicates candidate genes for genetic modification and molecular breeding in thermotolerance improvement.

## Introduction

Sessile plants constantly experience daily and seasonal fluctuations of environmental temperatures in nature. The ability to retain “a memory” or “stress imprint” of prior exposure to certain priming conditions for a certain length of time can make a plant more tolerant to future stress (Bruce et al., 2007; Wu et al., 2013; Martinez-Medina et al., 2016). Defense priming is an adaptive trait for promoting the plant to a persistently primed state of defense readiness for unpredictable environments, resulting in enhanced pest and disease resistance and abiotic stress tolerance (Conrath et al., 2006; Walter et al., 2013; Martinez-Medina et al., 2016). The primed memory can be induced by various natural and synthetic compounds, as well as biotic and abiotic stimuli (Conrath et al., 2006; Conrath et al., 2015). In the primed state, plants have some costs for storage of priming information but will have better performance during the subsequent stress, that is, priming enhanced plant benefit (Martinez-Medina et al., 2016). Moreover, frequent recurring stress memory can extend into future generations (Ramirez-Carrasco et al., 2017), which may be an evolutionary force for plants adapting to rugged environments. Therefore, it is necessary to investigate the mechanisms involved in stress memory and apply it into tolerance improvement or genetic modification for cultivar breeding by regulation of plant natural defense system.

With exposure to moderately elevated temperatures, plants can obtain acquired thermotolerance against subsequent otherwise lethal heat stress (HS), which is termed as heat acclimation (Wu et al., 2013). During this process, plants trigger the genetically reprogrammed heat shock response that activates multiple protection mechanisms. The main defense cascades that have been identified contain heat stress transcription factors (HSFs), which regulate heat shock proteins (HSPs) for protein folding and protection and some antioxidative or metabolic enzymes, such as ascorbate peroxidase 2 (APX2) and galactinol synthase 1 (GOLS1) (Mueller et al., 2015). However, after returning to control temperature after heat acclimation, how plants retain the memory of prior acclimation in the case of recurring HS has not been well understood. HSFA2 has been proven to be a heat-inducible transactivator sustaining the expression of heat-stress-associated 32-kDa protein coding gene (*HSA32*) and class I small HSP genes (*sHSPs*) to extend the duration of acquired thermotolerance after a long recovery period (>24 h) in Arabidopsis (Charng et al., 2006; Charng et al., 2007). A subsequent study demonstrated that a positive feedback loop between HSP101 and HSA32 helps to prolong heat acclimation memory (Wu et al., 2013). In addition, the upstream regulation mechanisms, including *miR156* and H3K4 methylation, were also investigated to determine their involvement in heat stress memory (Bäurle, 2016; Liu et al., 2018a). However, these results need more studies to verify, and whether other factors participate in heat memory and how memory is sustained in woody plants warrants further investigation.

Photosystem is the most sensitive component in response to HS. Inhibition of photosynthesis by HS is primarily attributable to the inactivation of ribulose-1,5-bisphosphate carboxylase/oxygenase (Rubisco), the central enzyme of the Calvin cycle for carbon fixation during photosynthesis (Crafts-Brandner and Salvucci, 2000; Kim and Portis Jr, 2005; Niinemets et al., 2017). The inactivation of Rubisco does not result from its own thermal instability but rather from that of its activator, Rubisco activase (RCA) (Portis, 2003; Salvucci and Crafts-Brandner, 2004). RCA is a nuclear-encoded chloroplast protein that is observed in many photosynthetic organisms. RCA belongs to the AAA+ chaperonin family and repairs Rubisco by removing inhibitory sugar phosphates from the active sites to maintain Rubisco in an active conformation (Bracher et al., 2017). Arabidopsis with modified thermostable *RCA* improves photosynthesis and growth rates under moderate HS compared with the lines expressing wild-type *RCA* (Kurek et al., 2007). In contrast, silencing of *RCA* increased the heat sensitivity of photosynthesis, photochemical quantum yield, and Rubisco activation state in Arabidopsis and tobacco (Sharkey et al., 2001; Salvucci, 2008). These studies provide evidence that the thermal stability of RCA is a major factor limiting plant photosynthesis under HS, but whether RCA participates in heat acclimation memory has not been determined.

Azaleas, which belong to the genus *Rhododendron*, are well-known woody ornamental plants widely used in landscaping and as houseplants around the world (De Riek et al., 2018). However, wild azalea species are largely distributed in cool environmental conditions, and the majority of azaleas that are now cultivated display poor ornamental effects in hot-summer regions (Geng et al., 2019). High temperature is the primary obstacle affecting the landscape application of azaleas and will become even more relevant with global warming. In view of the rich diversity of azalea species, exploring heat-tolerant germplasm and illuminating the heat response mechanisms for cultivar breeding is an imperative. In our study, a heat-tolerant wild germplasm *Rhododendron hainanense* Merr. (Subgen. *Tsutsusi*) was investigated to elucidate the mechanisms of heat response. *R. hainanense* natively distributes at an altitude of 200 to 900 m in Hainan, the southernmost province of China, and has strong adaptability to high temperature environments (Liu et al., 2018b). We found that heat acclimation with a 2-d recovery to control temperature still can confer acquired thermotolerance to *R. hainanense* against subsequent severe HS, which displays defense memory during the recovery period. The objectives of this study were to characterize the mechanisms and key factors involved in the heat acclimation memory by combined transcriptomic, proteomic, and physiological analyses. Prolonged protection of photosynthetic apparatus by upregulated chaperonins during the recovery period was identified to be critical for the defense memory, and a potential role of the thermostable RCA in photosynthetic acclimation to HS was specifically discussed.

## Materials and Methods

### Plant Growth Conditions and Heat Treatments

Cutting seedlings of *R. hainanense*, collected from Ornamental Germplasm Resource Nursery of Zhejiang University, were cultivated in a greenhouse with rough temperature control in the range of 15°C to 30°C throughout the year. Uniform two-year-old cutting seedlings were transferred into growth chambers at control temperature (25/18°C, day/night) with 65% relative humidity and a 14-h photoperiod with 90 µmol photons m^−2^ s^−1^ photosynthetically active radiation (PAR) for one month prior to treatments.

**Figure 1:**
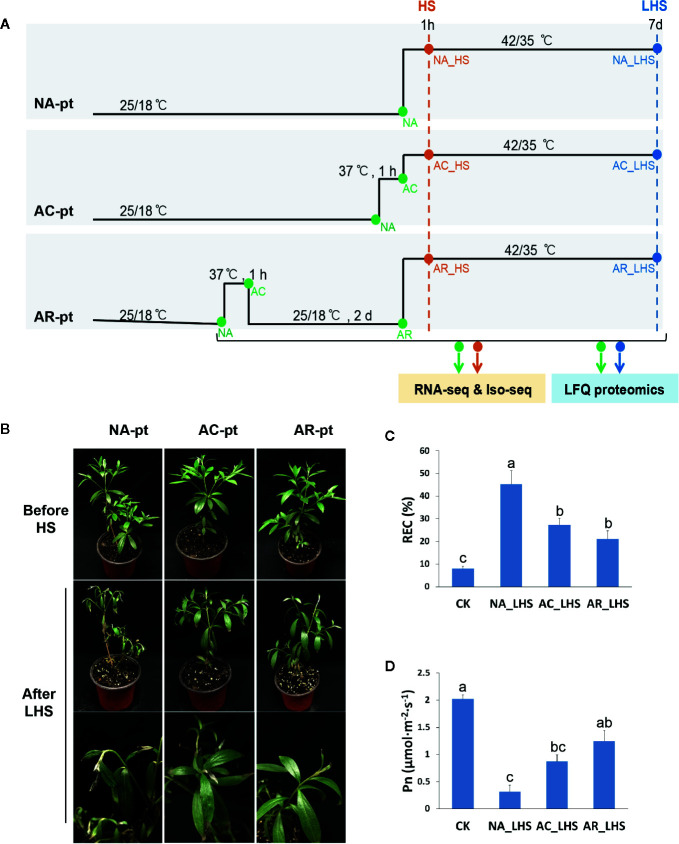
Effects of different pretreatments on *R. hainanense* under heat stress. **(A)** Sketch map of the different pretreatments (pt), heat stress, and sampling time points. NA, non-acclimation; AC, acclimation; AR, acclimation with recovery; HS, heat stress; LHS, long-term heat stress. Green and orange dots indicate samples performed RNA-seq and Iso-seq. Green and blue dots indicate samples performed label-free quantification (LFQ) proteomics. **(B)** Plant phenotypes before HS and after a 7-d LHS. **(C)** Relative electrical conductivity (REC) and **(D)** net photosynthesis rate (Pn) of plants with different treatments. Control (CK), plants without any treatment. Data are expressed as the mean values ± (standard deviation) SD of three biological replicates. Lowercase letters above the columns indicate multiple comparisons among different samples conducted by Duncan test at a significance level of 0.05.

Plants were subjected to three different pretreatments before HS (42/35°C, day/night temperature) (**Figure 1A**): non-acclimation (NA-pt), acclimation (AC-t) (37°C, 1 h), and acclimation (37°C, 1 h) with a 2-d recovery to control temperature (AR-pt). There are three biological replicates for each treatment. Samples were collected before HS, after 1 h of HS and 7 d of long-term HS (LHS). The two to five fully expanded leaves from the top of one to two branches of each plant were sampled at each time point for a replicate. The samples that are marked with same names (NA in NA-pt, AC-pt, and AR-pt, AC in AC-pt and AR-pt) were mixed. Eighteen samples (including replicates) indicated by green and orange dots were analyzed for transcriptome quantification individually, and 18 samples indicated by green and blue dots were analyzed for proteome quantification. Six samples (replicates mixed) indicated by green and orange dots were used to construct individual SMRTbell libraries, and the libraries were pooled with equimolar ratios before isoform-level transcriptome sequencing (Iso-seq) was performed. Collected leaves were flash frozen in liquid nitrogen and stored at −80°C.

### Physiological Measurements

The leaf net photosynthetic rate (Pn) was measured using a LI-6400 portable photosynthesis system (LI-COR, Lincoln, NE, USA), with parameters of 500 flow, atmospheric CO_2_ concentration, temperature at 25°C and PAR at 90 µmol photons m^−2^ s^−1^. Chlorophyll fluorescence, displayed as an Fv/Fm value, was detected by an Imaging-PAM chlorophyll fluorometer (Walz, Effeltrich, Germany). Plants were dark-adapted for 20 min before measurements, and the same position of each leaf was measured. Cellular membrane stability was estimated based on relative electrical conductivity (REC) (Blum and Ebercon, 1981). The initial (C_ini_) and maximum (C_max_) levels of REC were measured using a conductance meter (Thermo Scientific, Beverly, MA, USA). REC was calculated as REC (%) = (C_ini_/C_max_) × 100. Rubisco activation state was determined according to a previous method (Xu et al., 2013).

### Chloroplast Ultrastructure Observation

For chloroplast ultrastructure observation by transmission electron microscopy, leaf samples measuring 1 mm^2^ were fixed with 2.5% glutaraldehyde, embedded with resin, sliced by a Leica EM UC7 ultramicrotome (Leica Microsystems, Nussloch, Germany) and subjected to image acquisition with a HT7700 120KV transmission electron microscope (Hitachi, Tokyo, Japan).

### Iso-Seq Analysis

Total RNA from azalea leaves was extracted using the RNAprep Pure Plant Plus Kit (Polysaccharides & Polyphenolics-rich) (TIANGEN, Beijing, China). Poly(A) RNA (mRNA) was enriched by oligo(dT) magnetic beads and quantified using the Agilent 2100 Bioanalyzer. Full-length first-strand cDNA was synthesized using a UMI base PCR cDNA Synthesis Kit (BGI, Shenzhen, China). After large-scale amplification, cDNA was used to construct two SMRT cell libraries (0–5 K and 4.5–10 K) using a DNA Template Prep Kit (Pacific Biosciences of California). SMRT sequencing was carried out on the Pacific Bioscience Sequel platform. Subreads were filtered to obtain high-quality consensus transcripts using the SMRT Analysis Server. Blastx (Altschul et al., 1990) or Diamond (Buchfink et al., 2015) was used for NR, KOG, KEGG, and Swiss-Prot annotation. The Swiss-Prot database version used for protein search is release 2018_08, and taxonomy used for protein search is Viridiplantae. Release 2018_08 of Swiss-Prot contains 558125 sequence entries, comprising 200328830 amino acids. Blast2GO (Conesa et al., 2005) with NR annotation results was used for GO annotation.

### Transcriptome Quantification by RNA-Seq

Messenger RNA was obtained according to the method mentioned above, and fragmentation buffer was added to break mRNA into fragments, which were used as templates to synthesize first-strand cDNA. After second-strand cDNA synthesis, the fragments were purified and subjected to cohesive end repair, “A” addition and adapter ligation. Then, the suitable size of fragments was amplified by PCR. The mRNA libraries were quantified using the Agilent 2100 Bioanalyzer and sequenced on an Illumina HiSeq 4000 platform.

Raw reads were filtered using SOAPnuke software, and clean reads were obtained. Mapping of clean reads to the full-length transcriptome sequences was performed with Bowtie2 (Langmead and Salzberg, 2012), and transcript quantification (FPKM, Fragments Per Kilobase per Million) was calculated using RSEM (Li and Dewey, 2011). Transcript clusters with a time course were obtained with the software package Mfuzz (Kumar and Futschik, 2007). Transcripts with fold changes ≥ 2 and Q values ≤ 0.001 were identified as DETs using the R package DEGseq (Wang et al., 2009). Hierarchical cluster analysis was carried out with the pheatmap function of R software. Enrichment analysis of GO and KEGG terms was performed with the phyper function.

### Label-Free Quantification (LFQ) of Proteomics by SWATH-MS

Approximately 0.5 g of leaves were used for protein extraction as described previously (Zeng et al., 2017). Protein concentration was determined using a Bradford assay (Bradford, 1976), and protein quality was detected using SDS-PAGE. Trypsin digestion was performed (enzyme/protein = 1:40 w/w) overnight at 37°C using 100 μg protein for each sample. Peptides were separated on a liquid phase system UltiMate 3000 UHPLC (Thermo Fisher Scientific, San Jose, CA, USA) with a flow rate of 500 nl/min. The SWATH-MS spectral ion library was first generated with mixed samples using data-dependent acquisition (DDA) on a mass spectrometer Q-Exactive HF (Thermo Fisher Scientific). Individual samples were detected with data-independent acquisition (DIA) mode.

MaxQuant (Cox and Mann, 2008) was used for the identification of DDA data, and information satisfying FDR ≤ 1% will be used to establish the final spectral library. ProteinPilot 4.5 (Sciex) was used to search all of the DDA data thoroughly against the UniProt Swiss-Prot protein database to generate a spectral library. DIA data were quantified with Spectronaut (Bruderer et al., 2015), and differentially abundant proteins (DAPs) at fold change ≥ 2 and P value < 0.05 were identified and enriched using MSstats (Choi et al., 2014). PPI analysis was performed using the STRING database (von Mering et al., 2005), and the first 100 credibility of the interaction relation was selected to draw the network interaction graph. Prediction of subcellular localization of proteins was carried out with WoLF PSORT (Horton et al., 2007).

### qRT-PCR Analysis

Total RNA was used to synthesize the first-strand cDNA with the PrimeScript RT Reagent Kit with gDNA Eraser (Perfect Real Time) (Takara, Otsu, Japan). PCR was performed with TB Green^®^ Premix Ex Taq (Takara) on a CFX Connect Real-Time System (Bio-Rad, Hercules, CA, USA). Each sample was performed with three biological replicates. The relative expression level of genes was determined by the 2^−ΔΔCT^ method (Livak and Schmittgen, 2001) using 18S rRNA as the respective reference gene. Primers for qRT-PCR are listed in **Supplementary Table S1**.

### Immunoblot Assay

Total protein was prepared as mentioned above. The amino acid residues (QAPMDSGTHYAVM, 98–110 aa) representing the antigenic peptide were used to generate an anti-RCA1 rabbit peptide antibody. The commercial antibody anti-HSP21 and anti-GAPDH were ordered from Abcam and Proteintech, respectively. 30 μg of total soluble protein for each lane were separated on 12% SDS-PAGE and blotted 1 h to PVDF. Blots were blocked with 5% non-fat milk in TBST for 1 h at room temperature with agitation. Blot was incubated in the primary antibody at a dilution of 1: 4 000 overnight at 4°C with agitation. The second antibody (HRP-conjugated Affinipure Goat Anti-Rabbit IgG, Proteintech) was diluted to 1:8 000 in blocking solution for use. The blot was developed for 5 min with chemiluminescent detection reagent before image capture using a CCD imager (ChemiDoc MP).

### Firefly Luciferase Complementation Imaging Assay

ORFs of *RCA1-X1*, *Lhca2*, and *CCT3* were amplified from cDNA of *R. hainanense* leaves. ORF lacking stop codon of *RCA1-X1* was cloned into pCAMBIA1300-nLUC, and ORFs with stop codons of *Lhca2* and *CCT3* were cloned into pCAMBIA1300-cLUC, respectively. Constructs were sequenced to confirm accurate fusion and then introduced into *Agrobacterium tumefaciens* strain GV3101. Equal bacterial volumes of each construct (OD_600_ = 1.0) were mixed before co-infiltration into *Nicotiana benthamiana* leaves. After infiltration, *N. benthamiana* plants were cultivated for 60 h, and the infiltrated leaves then were smeared with one millimolar luciferin. The plants were kept in dark for 5 min before capturing the LUC image by a CCD imaging apparatus. Each assay consisted of at least three replicates.

### Statistical Analysis

Physiological data and protein levels of different samples were analyzed using the analysis of variance (ANOVA). Means were compared by the Duncan test at a significance level of 0.05 with SPSS statistical program (IBM Corporation, Armonk, New York, USA).

### Data Availability

The transcriptome raw data files were submitted to the Sequence Read Archive (SRA) database with the accession number PRJNA579430. GenBank accession numbers for alternative splicing transcripts of RCA genes are MN729585–MN729594. The mass spectrometry proteomics data have been deposited to the ProteomeXchange Consortium *via* the PRIDE (Perez-Riverol et al., 2019) partner repository with the dataset identifier PXD017005.

## Results

### Effects of Different Pretreatments on *R. hainanense* Against Heat Stress

To characterize defense responses to heat acclimation in the thermotolerant azalea *R. hainanense*, three groups of plants were exposed to non-acclimation pretreatment (NA-pt), acclimation pretreatment at 37°C for 1 h (AC-pt), and acclimation with a 2-d recovery pretreatment (AR-pt) (**Figure 1A**), respectively, before HS (42/35°C, day/night). Plants exhibited no changes in phenotype after pretreatments (**Figure 1B**). After 7 d of LHS, NA-pt plants showed severe injury with drooping shoot apex and withered leaves, while AC-pt and AR-pt plants displayed slight damage on the leaf tip. REC, as an indicator of membrane damage, increased after LHS in all treatments, and has a significantly higher level in NA-pt plants than in AC-pt and AR-pt plants (**Figure 1C**). Consistently, net photosynthetic rate (Pn) decreased after LHS and NA-pt plants had a lower level compared to AR-pt plants (**Figure 1D**). The phenotype and physiological measurements of *R. hainanense* after LHS demonstrate acquired thermotolerance through heat acclimation for AC-pt and AR-pt plants. Interestingly, a 2-d recovery to control temperature did not weaken but even enhance the acquired thermotolerance effects, which reveals an extension (or memory) of acquired thermotolerance during the recovery period.

### Transcriptome and Proteome Profiles After 1 h of Heat Acclimation

In order to investigate the mechanisms involved in the extension of acquired thermotolerance during the recovery period, we performed the combined omics analysis including RNA-seq and SWATH-MS-based LFQ proteomics for different time points, as shown in **Figure 1A**. A single-molecule long-read sequencing analysis, or termed Iso-seq, was performed as reference. We quantified 76,278 transcripts and 5,512 proteins in the transcriptome and proteome, respectively, and 5,402 members were correlated in both omics. The quantified numbers of molecules in both omics and their high-correlation ratios displayed high-quality of the sequencing data. Differentially expressed transcripts (DETs, **Supplementary Figure S1A, Data S1**) and proteins (DAPs, **Supplementary Figure S1B, Data S2**) of HS and LHS samples are higher than that of AC and AR, and downregulated molecules have more abundance compared to upregulated molecules in all comparisons.

**Figure 2:**
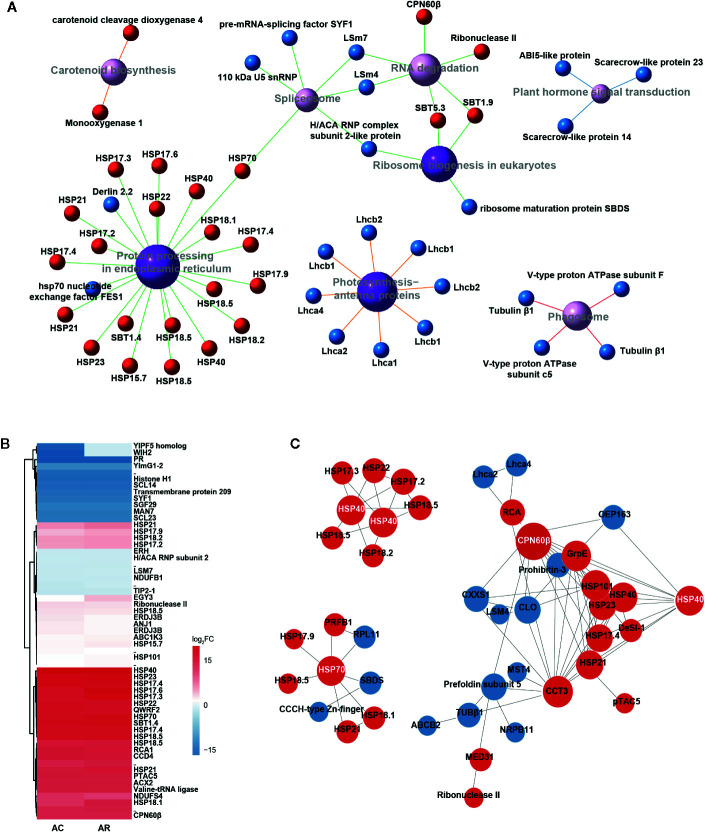
Identification and characteristic of DAPs in AC/NA and AR/NA. **(A)** KEGG pathway enrichment of DAPs in comparison of AC/NA. Red and blue balls represent up- and downregulated proteins, respectively. Purple balls indicate top eight enriched pathways, with dark color meaning significantly enriched and light color meaning enriched but not significantly, and larger areas indicate higher levels of enrichment. Different colors of line represent different classifications of pathway: red line indicates “cellular processes,” blue line indicates “environmental information processing,” green line indicates “genetic information processing” and orange line indicates “metabolism.” The detailed information of the DAPs was listed in **Supplementary Data S2**. **(B)** Heat map of expression patterns of DAPs in the intersection of AC/NA and AR/NA. The protein values are the averages from three biological replicates, normalized to NA, and then log_2_ transformed. The detailed information of the DAPs was listed in **Supplementary Data S3**. **(C)** Protein-protein interaction (PPI) analysis of DAPs in the union set of AC/NA and AR/NA. Red and blue circles represent up- and downregulated proteins, respectively. The size of the circle indicates the intensity of relationships, and the protein names that highlighted with white font indicate the high molecular chaperonins that tend to be network nodes. The detailed information of the DAPs was listed in **Supplementary Data S4**.

After 1 h of heat acclimation at 37°C, reprogramming of transcriptomic and proteomic level had already occurred. To identify the transcripts and proteins involved in heat acclimation, the KEGG pathway network of DETs and DAPs in AC/NA was employed to investigate primary pathways involved in heat acclimation. In both transcriptome and proteome, the most significantly enriched pathway is “protein processing in endoplasmic reticulum” (**Supplementary Figure S2**, **Figure 2A**), which is correlated with large amounts of upregulated proteins annotated as HSPs (**Supplementary Data S2**). The molecular weights of these HSPs range from 15 to 70 kDa, and most are in the range of 15 to 23 kDa, that is, the sHSPs. Another significantly enriched pathway is “photosynthesis-antenna proteins,” and the related DAPs are all downregulated light-harvesting complex chlorophyll a/b binding proteins (Lhca/b), which are key components in light harvesting of photosynthesis. In eukaryotic orthologous group (KOG) analysis (**Supplementary Figure S3A**), the largest number of 40 DAPs, were classified to the group of “posttranscriptional modification, protein turnover, chaperonins.” Moreover, it has the largest ratio of the DAPs localized in chloroplast (**Supplementary Figure S3B**).

### Transcriptome and Proteome Profiles After the 2-d Recovery

After the 2-d recovery following heat acclimation, “Protein processing in endoplasmic reticulum” remains the most enriched pathway in transcriptome (**Supplementary Figure S4A**) and proteome (**Supplementary Figure S4B**), and is correlated with numerous upregulated sHSPs (**Supplementary Data S2**). This result indicates that the sHSPs were upregulated in response to 1 h of heat acclimation and continuously maintained high levels during recovery to control temperature for 2 d. In addition, the pathway of “carotenoid biosynthesis” with 2/3 upregulated proteins and “cutin, suberine, and wax biosyntheses” with 2 downregulated proteins were significantly enriched in the comparison of AR/NA (**Supplementary Figure S4B**).

The DAP clustered heat map of the intersection between AC/NA and AR/NA shows the proteins that had continuous expression in heat acclimation and the following 2 d of recovery (**Figure 2B**, **Supplementary Data S3**). Accumulation levels of these proteins did not change much between AC and AR, and some proteins even have higher expression levels in AR compared to AC. In addition to the well-known HSPs, other proteins, such as QWRF motif-containing protein 2 (QWRF2), subtilisin-like protease 1.4 (SBT1.4), Rubisco activase (RCA), beta-subunit of chaperonin-60 (CPN60β), carotenoid cleavage dioxygenase 4 (CCD4) and plastid transcriptionally active chromosome 5 (pTAC5), also have high abundance. According to the protein and protein interaction (PPI) analysis (**Figure 2C**, **Supplementary Data S4**), there are lots of predicted interactions among the HSPs, which indicates their diversified binding patterns. It is worth noting that chaperonins with large molecular weights, such as HSP40, HSP70, and CPN60β, tend to be network nodes to interact with numerous sHSPs. In addition to the interactions with CPN60β, RCA was predicted to interact with a chaperonin, t-complex protein 1 subunit gamma (CCT3), and chlorophyll a binding protein Lhca2 and Lhca4. The continuous expressions of these proteins after the 2-d recovery probably play important roles in the extension of heat acclimation, and they would exhibit faster responses to the subsequent HS in AC-pt and AR-pt compared to NA-pt plants.

### Essential Factors Involved in Differences Between AR-pt and NA-pt Plants in Early Response to HS

**Figure 3:**
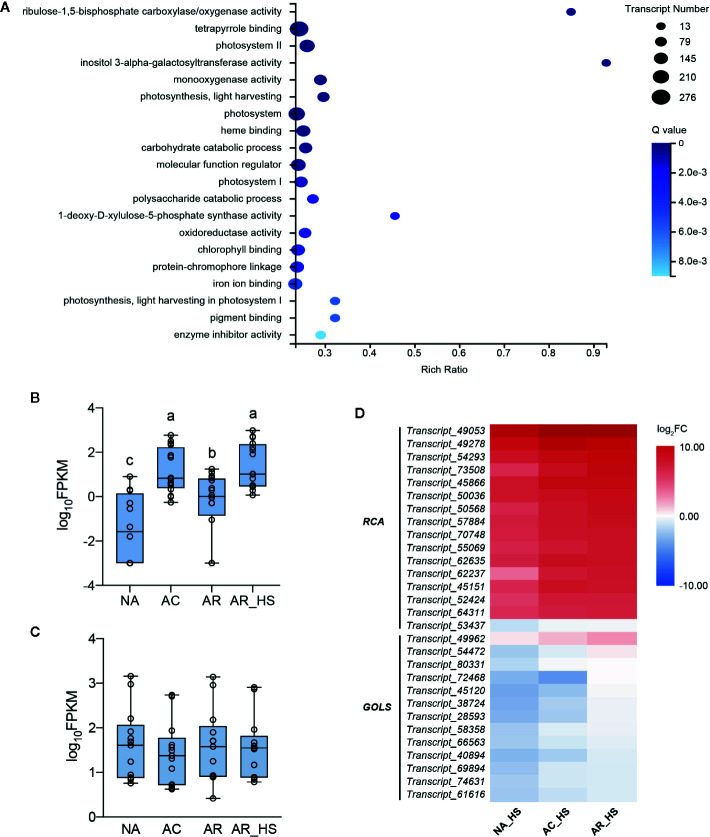
Transcriptional analysis of essential factors in the early stage of HS. **(A)** GO term enrichment of the DETs of AR_HS/NA_HS. Rich Ratio = term transcript number of selected transcript set/term transcript number of this species. Expression patterns of the **(B)** *RCA* transcripts in the GO terms of “ribulose-1,5-bisphosphate carboxylase/oxygenase activity” and **(C)** *GOLS* transcripts in “inositol 3-alpha-galactosyltransferase activity” in AR-pt plants. The data shown are log_10_ transformed FPKM values. Lowercase letters above the boxes indicate multiple comparisons among different samples conducted by Duncan test at a significance level of 0.05. **(D)** Heat map analysis of the *RCA* and *GOLS* profiles in NA_HS, AC_HS, and AR_HS samples. The values shown are the averages from three biological replicates, normalized to NA, and then log_2_ transformed. The detailed information of the DETs was listed in **Supplementary Data S5**.

Different early responses to HS can reveal the effects of different pretreatments and lead to divergent plant fates. A comparative transcriptome of AR_HS/NA_HS was investigated to clarify the essential factors involved in determining different fates of AR-pt and NA-pt plants. A total of 13,494 DETs of AR_HS/NA_HS were obtained and subjected to GO term analysis (**Figure 3A**). The terms “ribulose-1,5-bisphosphate carboxylase/oxygenase activity” and “inositol 3-alpha-galactosyltransferase activity,” annotated as 16 *RCAs* and 13 *GOLSs*, respectively, were significantly enriched. Moreover, these two terms had very high rich ratio, more than 85%, which means that most of the transcripts annotated in the two terms are involved in the different fate of AR-pt and NA-pt plants.

During the pre-treatment of AR plants, FPKM values of the 16 *RCA* and 13 *GOLS* transcripts were analyzed to display their expression patterns. Interestingly, transcript levels of *RCAs* were induced by incubation at 37°C, decreased but not to initial levels after 2 d recovery, and increasing transcript levels were detected after heat shock (**Figure 3B**). This indicated that these *RCA* transcripts have low expression at control temperature and are heat inducible upon elevated temperatures. However, the abundance of *GOLS* transcripts had no significant changes during the pretreatment of AR plants (**Figure 3C**). In addition, *RCA* transcripts (except Transcript_53437) had higher elevated levels than *GOLSs* in all three groups of plants in early response to HS (**Figure 3D**, **Supplementary Data S5**).

### Expression Patterns of Photosynthesis-Related Genes

**Figure 4:**
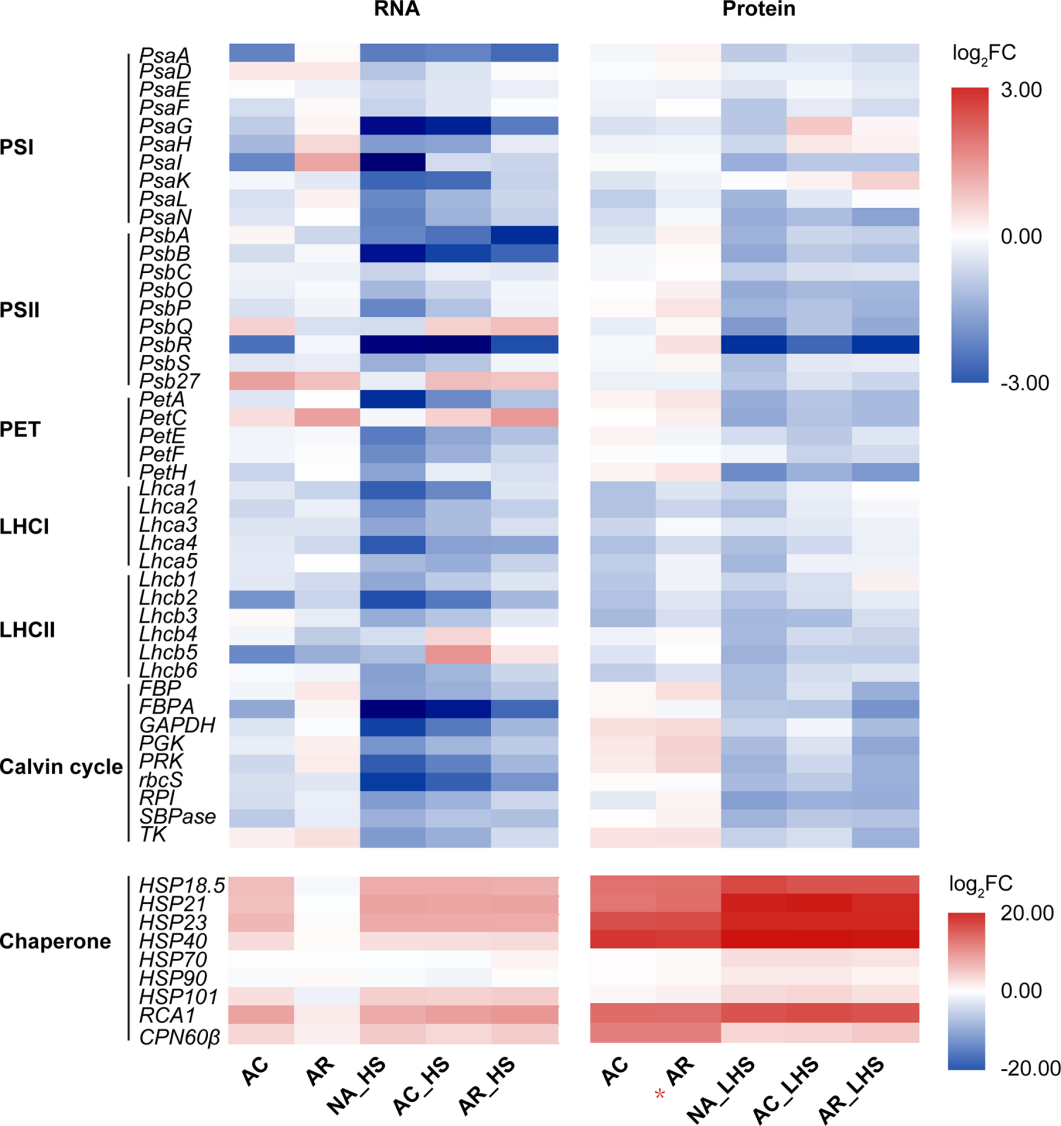
Expression analysis of photosynthesis-related genes at RNA and protein levels. Genes are grouped in the functional categories PSI (photosystem I), PSII, PET (photosynthetic electron transport), LHCI (light-harvesting complex I), LHCII, Calvin cycle, and chloroplast-localized chaperonin. The top panel and bottom panel use different scale legend. The values shown are the averages from three biological replicates, normalized to NA, and then log_2_ transformed. The asterisk (*) indicates the key column that chaperonins sustainably accumulated at high levels. The detailed information on the photosynthesis-related genes was listed in **Supplementary Data S6**.

In the GO analysis of AR_HS/NA_HS (**Figure 3A**), besides “ribulose-1,5-bisphosphate carboxylase/oxygenase activity” and “inositol 3-alpha-galactosyltransferase activity,” other significantly enriched GO terms are most associated with photosynthesis, which indicates potential differences of this process between AR-pt and NA-pt plants. We performed expression analysis of photosynthesis-related genes and the chloroplast-localized chaperonins at both RNA and protein levels (**Figure 4**, **Supplementary Data S6**). From the heat map, we can see that photosynthesis-related genes are most downregulated by AC and the both light-harvesting complex (LHCI and LHCII) genes decrease markedly at protein level. After AR, these genes also recovered close to initial levels in RNA and protein profiles, but proteins of photosystem II (PSII), photosynthetic electron transport (PET) and Calvin cycle had some accumulations. Moreover, these photosynthesis-related genes had a strong downregulation after early HS and LHS in all three groups of plants, but AR-pt plants had less decreases than NA-pt plants.

The chloroplast-localized chaperonins were most upregulated by AC and had significant accumulations at protein level (**Figure 4**, **Supplementary Data S6**). After AR, the chaperonins recovered to initial expressions at RNA level while maintained high accumulations at protein level, especially for *sHSPs*, *RCA1*, and *CPN60β*. The sustainable accumulation of these chaperonins in AR-pt plants before HS probably provide faster and earlier protection of photosynthesis-related proteins than NA-pt plants in response to the subsequent HS. As described earlier, *RCA1* had higher transcript levels in AR-pt plants compared to NA-pt in early HS, but other chaperonins did not show this difference. In addition, all chaperonins, except CPN60β, had greater protein abundance after 7 d of LHS.

### Protein Profiles and Damage Assessment of Photosynthetic Apparatus After 7 d of Heat Stress

After 7 d of LHS, the heat responsive proteins that highly accumulated in AC and AR had no significant difference among AR_LHS, NA_LHS, and AC_LHS (**Supplementary Figure S5A**, **Supplementary Data S7**). In the KEGG enrichment of AR_LHS/NA_LHS, some physiological metabolism pathways were enriched (**Supplementary Figure S5B**). The photosynthesis-antenna proteins, ABC transporters, proteins related to glucoronate metabolism and lipid metabolism had higher levels in AR-pt plants compared to NA-pt plants. Most proteins annotated in the “starch and sucrose metabolism” and “RNA degradation” pathways had lower levels in AR-pt plants. It is likely that the plants enter a relatively balanced state in the LHS and that AR-pt plants recover some normal physiological activities, such as light harvesting, substrate transport, and lipid metabolism. However, NA-pt plants may still struggle for survival and need more energy from starch and sucrose and need to deal with more RNA degradation caused by HS.

**Figure 5:**
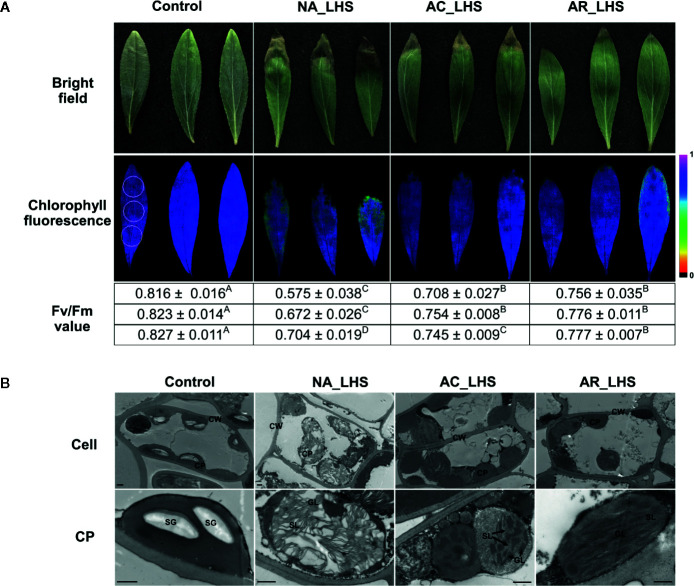
Damage analysis of differentially-pretreated plants after LHS. **(A)** Photochemical efficiency and **(B)** chloroplast ultrastructure observation of the leaves after LHS with non-stressed leaves as control. The fluorescence color indicates Fv/Fm value, and the three circles (1 cm in diameter) that marked in the control image represent detected regions (upper, middle, and lower part) for each leaf. Fv/Fm values of the three detected regions are correspondingly listed below the images, which are shown as mean value ± SD of three biological replicates. Superscript capital letters indicate multiple comparisons among different treatments of the same detected region, which was conducted by Duncan test at a significance level of 0.05. CP, chloroplast; CW, cell wall; SG, starch granule; GL, granum lamella; SL, stroma lamella. Bar indicates 10 μm.

In view of the major participation of photosystems in heat response, we detected maximum photochemical efficiency calculated as Fv/Fm value for monitoring heat effects on photosystem II, which has been considered the primary part of heat impairments on photosynthesis. Three regions (upper, middle, and lower part) including the healthy and necrotic portion of each leaf were detected (**Figure 5A**). Fv/Fm of all the portions significantly declined after LHS compared to control, with a greater degree in NA-pt than AC-pt and AR-pt plants. The seriously heat-damaged part of leaf tips was necrotic and did not have fluorescence signal, which was most obvious in NA-pt plants. For the chloroplast ultrastructure (**Figure 5B**), plants without HS have regular spindle-shaped and evenly distributed chloroplasts with many starch granules and compact granum lamella and stroma lamella. After LHS, the chloroplast number decreased, and the shape swelled to irregular rotund and oval shapes. The granum lamella and stroma lamella became loose, especially in NA-pt plants.

### Structure and Expression Analysis of the Heat-Induced RCA1 and the Constitutively Expressed RCA2 and RCA3

**Figure 6:**
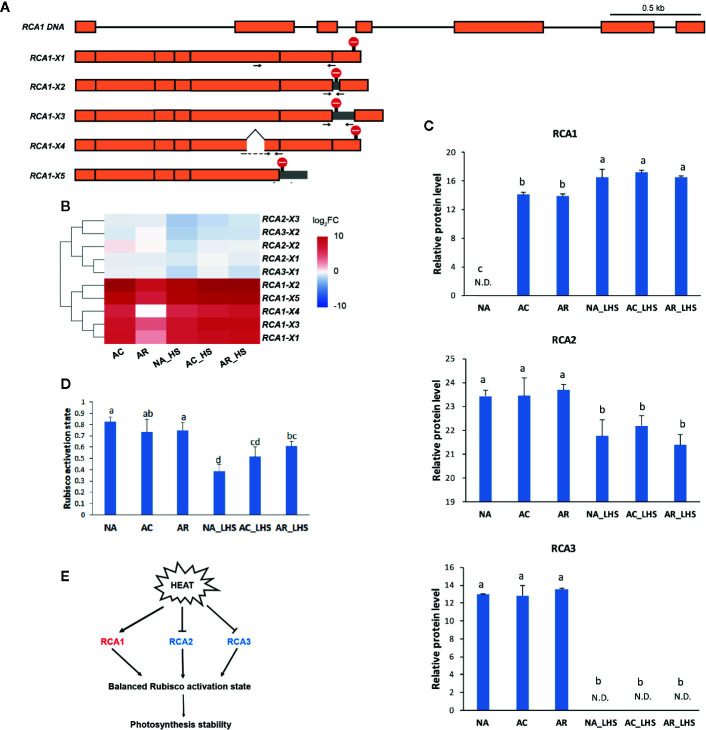
Structural and functional analysis of *RCA* genes in response to heat treatments. **(A)** Schematic diagram of the DNA and alternatively spliced transcripts (*X1*–*5*) of *RCA1*. Orange module represents exon and line represents intron. Stop sign indicates the position of termination codon. Gray module represents retained intron. Positions of specific primers for qRT-PCR are marked as arrows and primer sequences are listed in **Supplementary Table S1**. **(B)** Heat map of alternatively spliced transcripts of *RCA*. The values are averages from three biological replicates, normalized to NA, and then log_2_ transformed. **(C)** Protein expression level of *RCA* genes. The values of relative protein level were performed with median normalization and log_2_ transformation. N.D., not detected. **(D)** Rubisco activation state of the plants under different treatments. Lowercase letters above the columns indicate multiple comparisons among different samples conducted by Duncan test at a significance level of 0.05. **(E)** The regulation network that RCA involved. Heat induces RCA1 but inhibits RCA2 and RCA3, which results in a relative balanced Rubisco activation state and photosynthesis stability.

By comprehensive analysis of the transcriptome and proteome, RCA1, which is highly enriched in both omics and, especially in the early stage of HS, was selected for further investigation. To characterize all the *RCA* genes, all the *RCA* transcripts were identified from the Iso-seq sequence database and classified into three members (termed *RCA1*, *RCA2*, and *RCA3*) due to sequence identity and BLAST analysis with other species. Moreover, there are alternatively spliced transcripts for each *RCA* gene member: 5 for *RCA1*, 3 for *RCA2* and 2 for *RCA3* (**Figure 6A**, **Supplementary Figure S6**), and these transcripts were verified by cloning and sequencing with primers listed in **Supplementary Table S1**. Most alternatively spliced transcripts are introduced early termination codons, which make shorter coding regions.

To investigate the expression patterns of different *RCA* gene members, FPKM values (**Supplementary Figure S7**) and relative expression levels (**Figure 6B**) of different alternatively spliced transcripts were investigated. The major alternative splicing isoform of *RCA1* is *RCA1-X1*, which had the highest FPKM values under the stages tested (**Supplementary Figure S7A**). In NA and AR stage, all the *RCA1* isoforms almost had no expression, but under the heat-treated stages (AC, NA_HS, AC_HS, and AR_HS), *RCA1-X1* had much higher FPKM values than other *RCA1* isoforms. The major alternative splicing isoform of *RCA2* is *RCA2-X3*, whose expression was high before heat (NA) but decreased much after other treatments (**Supplementary Figure S7B**). Two isoforms of *RCA3* also expressed under control temperature and decreased after heat treatments (**Supplementary Figure S7B**). These results demonstrated that *RCA1* gene is heat inducible, and *RCA2* and *RCA3* are constitutively expressed. Relative levels of the alternatively spliced transcripts for the different time points compared to NA were identified and clustered in the heat map (**Figure 6B**). The upregulated transcripts all belong to *RCA1*, while *RCA2* and *RCA3* transcripts were generally not changed or slightly downregulated in response to heat. Moreover, all the RCA transcripts had higher levels in AR_HS compared to NA_HS (**Figure 6B**). We also designed specific primers to detect expression levels within 12 h of treatment at different temperatures by qRT-PCR (**Supplementary Figure S8**). The regulation patterns in response to heat are similar with RNA-seq data, and *RCA1* showed more intense upregulation and maintained longer expression under 42°C treatment compared to 37°C.

**Figure 7:**
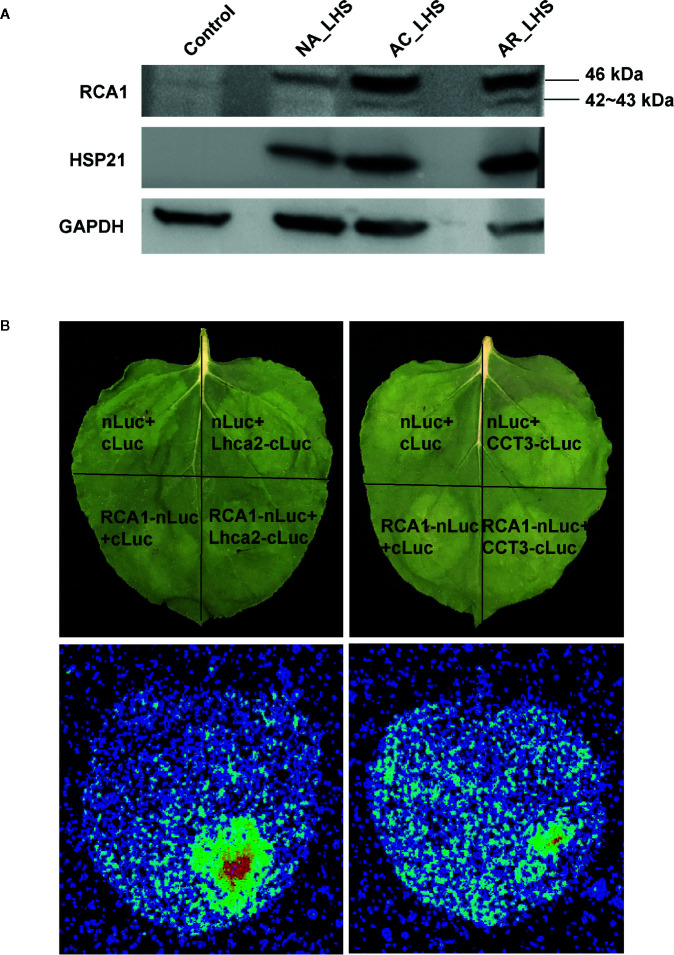
Validation of RCA1 thermostability and interacting proteins. **(A)** Immunoblot assay to confirm thermostability of RCA1. HSP21 was presented as a representative of HSPs and GAPDH as a loading control. **(B)** Firefly luciferase complementation imaging assay to confirm interacting proteins of RCA1. Partial Luc fusion constructs were transiently coexpressed in *N. benthamiana* leaves. Top panel indicate the partition for differentially transformed constructs. Luc signals, observed using a CCD camera, indicate protein interactions.

In proteomics, only one protein sequence was identified for each *RCA* gene member to quantify the protein expression level (**Figure 6C**), which may be due to technical limitations that cannot distinguish the highly similar protein sequences coded by the alternatively spliced transcripts or because some transcripts may be incapable of translating to proteins. RCA1 protein could not be detected at control temperature but was quickly induced by AC, was maintained high levels even after AR, and could be triggered to a greater degree after 7 d of LHS. However, RCA2 and RCA3 are constitutively expressed at control temperature, are not affected by AC and AR, but decreased to a great extent (RCA2) or completely degraded (RCA3) after LHS. Moreover, Rubisco activation state of all the plants decreased after LHS but has lower level in NA-pt plants compared to AR-pt (**Figure 6D**). Therefore, heat-induced thermostable RCA1 can supplement the depletion of RCA2 and RCA3, which maintains relatively balanced Rubisco activation state and photosynthesis stability under HS (**Figure 6E**). To confirm the thermostability of RCA1, immunoblot assay was performed (**Figure 7A**). After LHS, RCA1 protein had high accumulation in 46 kDa (RCA1-X1) and less in 42 to 43 kDa (probably RCA1-X2–4). The accumulation of RCA1 is similar to that of HSP21, which also had high abundance after LHS in proteomics analysis. Lhca2 and CCT3, which were predicted to have interactions with RCA in PPI analysis, were verified their interactions with RCA1 by firefly luciferase complementation imaging assays (**Figure 7B**).

## Discussion

### Protein-Level Sustainability of Chaperonins in the Recovery Period Contributes to Heat Acclimation Memory

**Figure 8:**
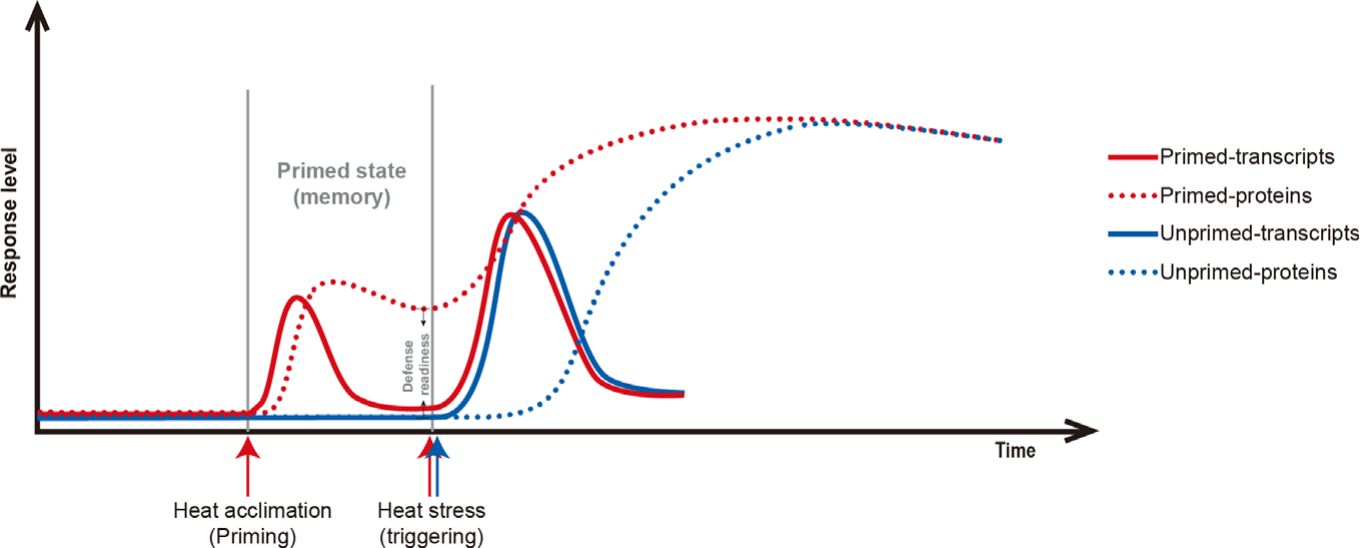
Scheme of the relations of chaperonin genes at transcript (solid line) and protein levels (dotted line) in primed (red line) versus unprimed (blue line) plants. Heat acclimation, as a priming stimulus, induces chaperonin genes at transient transcript level and sustained protein level. After priming, the status is term primed state or memory for storing reprogrammed information. The difference between primed- and unprimed-proteins are the defense readiness for primed plants. Upon subsequent HS, the chaperonins of primed plants are triggered faster and stronger protection than unprimed plants. After a certain of LHS, the transcript and protein levels of chaperonin genes have no difference between primed and unprimed plants, but the differences in the early stage of HS determine their final different performance.

Acclimation to fluctuating temperatures and possession of acclimation memory for unpredictable environmental challenges are crucial for plant survival. However, how plants possess the acclimation memory before the reoccurring stress is not well understood. In this study, heat acclimation can confer acquired thermotolerance to *R. hainanense* plants against subsequent severe HS, even when a 2-d recovery to control temperature separates the acclimation and stress periods. From the transcriptome and proteome analyses with physiological measurements, we figure out the response patterns of chaperonins at transcript and protein levels (**Figure 8**). Upon heat acclimation priming, transcript and protein levels of these chaperonins increased rapidly, and the plants were induced into a primed state. When recover to control temperature, transcripts decreased in short time while proteins sustained a prolonged protection at least for 2 d. The higher protein levels in primed than unprimed plants before HS are the defense readiness, which would provide faster and stronger protection in the early stage of subsequent HS. Although the proteins of unprimed plants had the similar levels as those of primed plants after 7 d of LHS, the different defense power in the early stage of HS determined the different performance of the two groups of plants. The primed plants need some costs for maintaining the reprogrammed primed state but have a better foundation of defense readiness for confronting HS. Therefore, the sustainably accumulated chaperonins in the recovery period are crucial for heat acclimation memory and extension of acquired thermotolerance.

Most of the chaperonins that accumulated in the recovery period are HSPs (**Figure 2B**, **Supplementary Figure S2**). Large amounts of the HSPs were predicted to localize in chloroplast and other cell components, and sHSPs, ranging from 15 to 23 kDa, have the largest ratio in these proteins. It has been known that unfolded proteins are easy to form large aggregates that severely impede normal cellular functions under HS, and the main function of HSPs is to bind the unfolded proteins to limit misfolding and resolve aggregates (Queitsch et al., 2000; Jacob et al., 2017; Muthusamy et al., 2017). Thus, the HSPs accumulated in the recovery period could play roles in maintaining proteome homeostasis with refolding or degrading diverse clients when the subsequent HS occurs. High-molecular-weight HSPs tend to be network nodes that connect numerous sHSPs to assemble multimeric complexes as homopolymers or heteropolymers, which is possibly because of the small size and flexibility of sHSPs, and it is conductive to switch on high-efficiency defense system in a short time (Lee and Vierling, 2000; Carra et al., 2017).

### Equipped Chaperonin Protection of Chloroplast in the Recovery Period Decreases Damage of Photosynthesis Under Subsequent HS

**Figure 9:**
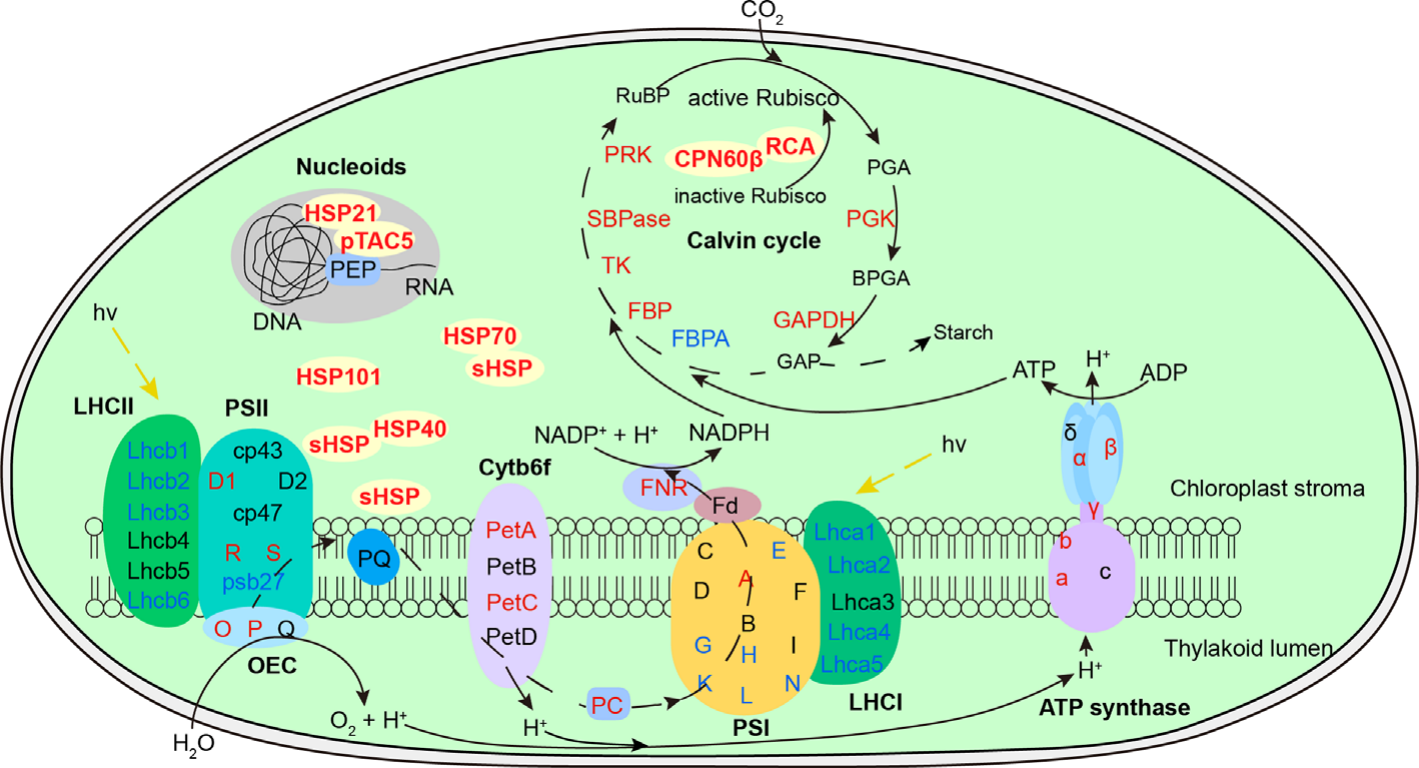
Defense readiness of chloroplast after 2-d recovery following heat acclimation. Different colors of protein names represent different protein level expression with red representing upregulation while blue as downregulation. The level of proteins was determined based on log_2_ fold change of AR compared to NA. After 2-d recovery, most subunits of both light-harvesting complex (LHC) were downregulated. Some subunits of photosynthetic electron transport, ATP synthase and Calvin cycle were upregulated. The significantly accumulated chaperonins provide equipped protection of photosystem, Rubisco activation, and plastid-encoded RNA polymerase (PEP) function against the subsequent HS.

Photosynthesis is considered most susceptible to high temperature stress among plant cell functions, and the primary sites of targets in heat exposure are photosystems (Allakhverdiev et al., 2008; Ahammed et al., 2018). In this study, photosynthesis is also significantly enriched in the heat acclimation memory, and the equipped status of chloroplast in the recovery period is critical for tolerance to the subsequent HS (**Figure 9**). Both LHCs were sensitive to heat signal and most chlorophyll a/b-binding proteins (Lhca1/2/4/5 in LHCI and Lhcb1/2/3/6 in LHCII) were reduced probably for cutting down light harvesting to prevent surplus light energy-caused damage. Moreover, some proteins of PET (PSII subunit O and P, PC and FNR), ATP synthase (subunit α, β, γ, a, b) and Calvin cycle (PGK, GAPDH, FBP, TK, SBPase, and PRK) were upregulated, which is possibly for accelerative consume of excess energy. The most important equipped components in the recovery period are the significantly accumulated chloroplast-localized chaperonins that could provide faster and stronger protection for photosystem in the early stage of HS. It has been demonstrated that sHSPs could associate with chloroplast thylakoids and protect oxygen-evolving complex (OEC) proteins of PSII against HS (Heckathorn et al., 1998). Variation in induced PET thermotolerance of five ecotypes of *Chenopodium album* was as well as highly correlated with chloroplast sHSP protection (Barua et al., 2003). And the interaction of HSP21 and pTAC5 is required for chloroplast development under HS by maintaining plastid-encoded RNA polymerase (PEP) function (Zhong et al., 2013). RCA plays important roles in maintaining the active state of Rubisco to ensure photosynthesis, and CPN60β was reported to associate with RCA to protect photosynthesis during HS (Salvucci, 2008). In this study, CPN60β possibly protect RCA2 rather than RCA1 because the protein content of CPN60β after LHS was decreased similar to changes of RCA2 (**Figure 4**). RCA1 could interact with Lhca2 according to the firefly luciferase complementation imaging assays (**Figure 7B**), which implies that RCA may affect light harvesting of LHCI in addition to carbon fixation in photosynthesis. These diverse functions of the chaperonins demonstrate that it is well equipped for AR-pt plants in the primed state before HS. And the results of Fv/Fm and chloroplast ultrastructure after LHS (**Figure 5**) indicate that the acclimated defense readiness enhanced thermostability of the photosynthetic apparatus.

### RCA1 Probably Play Vital Roles in the Photosynthetic Acclimation to High Temperature

It has been proposed that reduced photosynthesis at high temperature is probably due to Rubisco inactivation caused by thermal lability of RCA (Crafts-Brandner and Law, 2000). However, the protein contents of total RCA were increased in the heat acclimation and recovery periods owning to quickly heat-induced RCA1 in *R. hainanense* (**Figure 6C**). Thus, the key factor of Rubisco activation for photosynthesis was not inhibited but improved after heat acclimation. The heat-induced RCA1 has the characteristics of quick and strong response to heat treatment, which is totally different with the constitutively expressed RCA2 and RCA3 but similar to HSPs. Heat-induced RCA is not pervasive in plants but only found in a few of species. It was first identified in maize and verified as a molecular chaperonin rather than a conventional enzyme (Sanchez de Jimenez et al., 1995). In wheat, HS increased the accumulation of the constitutive 42-kDa RCA and induced the synthesis of a putative 41-kDa form, and after 24 h of recovery from HS, the 42-kDa activase returned to control levels while a small amount of the 41-kDa protein was still expressed (Law and Crafts-Brandner, 2001). It was also demonstrated that the induction of a new form of RCA may constitute a mechanism of photosynthetic acclimation to HS in cotton (Law et al., 2001). Thus, heat-induced RCA is very important for confronting HS and needs further study in more plants.

In addition to the heat-inducible characteristic, thermostability is another significant feature of RCA1. After 7 d of LHS, RCA2, and RCA3 decreased or even totally degraded, while RCA1 displayed high thermostability to maintain relatively balanced Rubisco activity and photosynthesis (**Figures 6C, D**). These findings reveal the major role of thermostable RCA1, rather than RCA2 or RCA3, in the activation and protection of Rubisco or possibly other proteins during LHS in *R. hainanense*. Both RCA1 and HSPs were significantly accumulated in LHS, but *RCA1* rather than HSPs has a higher transcript level in AR-pt plants compared to NA-pt plants in the early stage of HS (**Figure 4**), which reflects the effects of accumulation. Moreover, elevated levels of induced and constitutive RCA proteins persist hours after expression of HSPs has decreased to control levels in both maize and wheat leaves (Law and Crafts-Brandner, 2001), which indicates that heat-increased RCA probably play more important roles than HSPs in the maintenance of heat memory. The heat-induced characteristics, sustainable expression in the recovery period and high thermostability of RCA1 indicate that it is a vital factor involved in photosynthetic acclimation to HS. The relationships between RCA1 and the reported heat memory associated factors, such as HSFA2, miRNA, or histone methylation, merit further research.

It is worth noting that RCA1 of *R. hainanense* has five alternatively spliced transcripts (**Figure 6A**), which could be translated to four protein isoforms with discrepant structures. Alternative splicing is a kind of posttranscriptional regulation and has a highly important role in expanding proteomic diversity and functional complexity in higher eukaryotes (Nilsen and Graveley, 2010; Carvalho et al., 2013). We cannot identify all alternative splicing events in response to HS without whole genome sequence of *R. hainanense*. But it has been demonstrated that alternative splicing is involved in most plant processes and is particularly prevalent in plants exposed to environmental stresses (Graveley, 2001). The effects of alternative splicing include the production of protein isoforms with various loss- or gain-of-function and/or posttranslational modification, altered subcellular localization, enzymatic activity or protein stability in various situations (Stamm et al., 2005), which provides additional adaptive advantages to the organisms. It is also reported alternative splicing of RCA genes in other plant species, but most generating two isoforms (Carmo-Silva et al., 2015; Nagarajan and Gill, 2018). The high ratio of alternative splicing of *RCA1* transcripts in *R. hainanense* reveals the functional complexity and importance of *RCA1* gene in heat tolerance. And the major isoform, *RCA1-X1*, probably plays more important roles than other *RCA1* isoforms. However, alternative splicing sometimes generates nonfunctional mRNAs, which usually contain premature termination codons and can be targeted for degradation by nonsense-mediated decay (NMD) (Wollerton et al., 2004; Carvalho et al., 2013). The transcript diversity of *RCA1* likely contributes to thermotolerance, or some transcripts might be degraded by NMD, which will be identified by their gain or loss of function in our future study.

## Conclusions

In summary, we found that a 2-d recovery to control temperature following heat acclimation did not weaken but even enhance the acquired thermotolerance in the heat-tolerant azalea *R. hainanense*. The sustainable accumulations of chaperonins at protein-level rather than transcriptional-level after 2-d recovery contribute to the extension of heat acclimation memory. And the most affected biological process is photosynthesis, which decreased less in AR-pt plants with the equipped protection of chloroplast-localized chaperonins. Heat-induced RCA1 has high thermostability under LHS and play important roles in balanced Rubisco activation state and photosynthetic acclimation to elevated temperature. Further studies will characterize the functional differences of alternative-spliced transcripts of *RCA1* and find the regulation factors that activate *RCA1*-mediated heat tolerance. In practical application, appropriate priming that can induce defense readiness, which may not be limited to heat acclimation, can be applied to stimulate the plant defense system for enhanced tolerance to upcoming HS. The chaperonins determined in our study can be used as indicators of primed defense readiness and as candidate genes to improve heat tolerance of azalea and other plants through genetic engineering.

## Data Availability Statement

The datasets presented in this study can be found in online repositories. The names of the repository/repositories and accession number(s) can be found in the article/**Supplementary Material**.

## Author Contributions

YX provided support of research expenses. XW, HZ, and YX conceived and designed the study. XW and ZL performed experiments and wrote the initial manuscript. XW, BL, and ME performed data analysis. All authors contributed to the article and approved the submitted version.

## Funding

This work was supported by the National Natural Science Foundation of China (grant number, 31901356), Postdoctoral Science Foundation of China (grant number, 2018M632484) and Zhejiang Science and Technology Major Program on Agricultural New Variety Breeding (grant number, 2016C02056-12).

## Conflict of Interest

The authors declare that the research was conducted in the absence of any commercial or financial relationships that could be construed as a potential conflict of interest.
