# Supplementary material for: Combined Proteome and Transcriptome Analysis of Heat-Primed Azalea Reveals New Insights Into Plant Heat Acclimation Memory
Source: Front Plant Sci. 2020 Aug 19;11:1278. doi: 10.3389/fpls.2020.01278 (PMC7466565; doi:10.3389/fpls.2020.01278)
Supplement: Supplementary file 2 [file Table_1.docx]

Supplementary Material

**Supplementary Table S1** Primers information. Primers for qRT-PCR are marked suffix as “rt” and for full length amplification as “fl”.

| **Primer Name** | | **Forward Primer  Sequence (5’-3’)** | **Reverse Primer  Sequence (5’-3’)** |
| --- | --- | --- | --- |
| 18S rRNA-rt  RCA1-X1-rt  RCA1-X2-rt  RCA1-X3-rt  RCA1-X4-rt  RCA1-X5-rt  RCA2-X1-rt  RCA2-X2-rt  RCA2-X3-rt  RCA3-X1-rt  RCA3-X2-rt  RCA1-X1~4-fl  RCA1-X5-fl  RCA2-fl  RCA3-X1-fl  RCA3-X2-fl | CGCATTCCCCACTGTATTAGAC  ACGTTGTATGCGCCCCTAAT  GACAAGCTCCTCGAGTACGG  GTACCTAAGCGAAGCAGCCC  TGTGGTCACTGGTATTTTCAGGA  CGGATTGGTGTTTGCACAGG  AGAACAGGAGAATGTGAAGAGA  GAGAGTCCAATTGGCCGACA  GAGTCCAATTGGCCGACAAG  GACGGCTAGCAAGTACCTGTG  ATGTGCCCCCAAAACAAACG  TCCACTTCGTCAGCTTTCTT  AGATCCCAAACAGACCCTTGAT  AATCACAGCCTACAGAGCAGG  TCGTCACTAATTTAAGACCAACGC  GACCAACGCCAAAATCAATGG | | CGTAACAAGGTTTCCGTAGGTG  GAGCTGCTTTGCCATAGAAAGTT  TGCTTTGCCTCCTGCTAAGG  TCTCAGGCTTTGTATCAGCCA  CCTTAGAGCGCCGAAGAAGT  TTTCATCATCTGGAGCGCCT  TGAGCTGCTTGACCATAGAA  CTGCTTGACCTTCGGGATCA  TCATTAGCAAACGAGGGGGAA  AAAGAGCAAGTGTCATCCCTCC  CAAAGCCATGAGCTTGCATGA  AGGGTCTCAACTTCACACATCC  ACCCACAAACGAGCCACATT  TACACAGCAGCAGTCACGAA  GAGCAAGTGTCATCCCTCCA  GCAACTTTTTGCAGTTTGCCC |
