# Supplementary material for: Combined Proteome and Transcriptome Analysis of Heat-Primed Azalea Reveals New Insights Into Plant Heat Acclimation Memory
Source: Front Plant Sci. 2020 Aug 19;11:1278. doi: 10.3389/fpls.2020.01278 (PMC7466565; doi:10.3389/fpls.2020.01278)
Supplement: Supplementary file 3 [file Image_1.pdf]

Supplementary Material

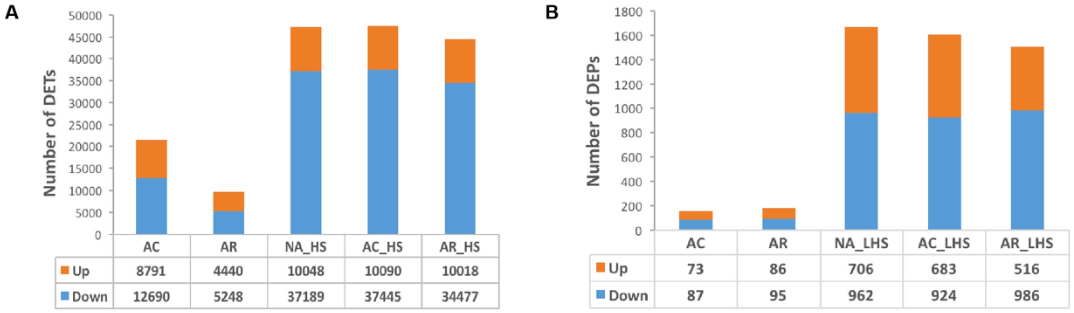

Supplementary Figure S1. Number of differentially expressed transcripts and proteins. **(A)** Number of up- and downregulated differentially expressed transcripts (DETs) of the time points compared to NA. Transcripts were considered differentially expressed at fold changes ≥ 2 and Q values ≤ 0.001. **(B)** Number of up- and downregulated differentially abundant proteins (DAPs) of the time points compared to NA. Proteins were considered differentially expressed at fold changes ≥ 2 and P values < 0.05.

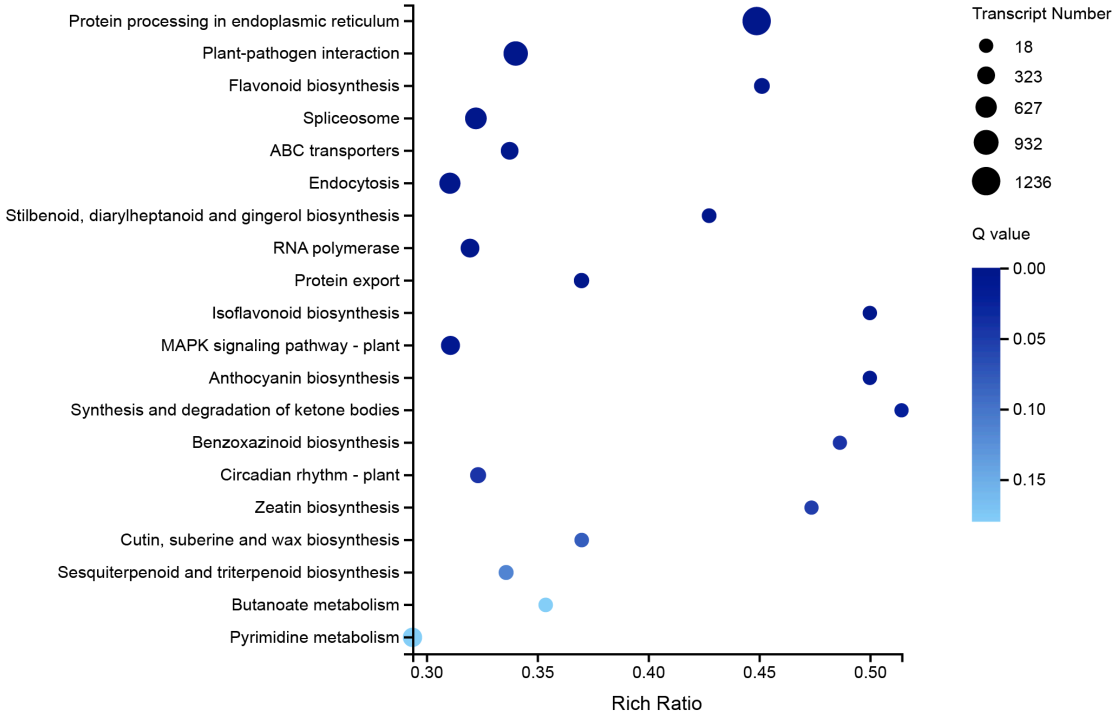

Supplementary Figure S2. KEGG pathway enrichment of DETs in AC/NA. Bubble size indicates transcript number and bubble color indicates Q value.

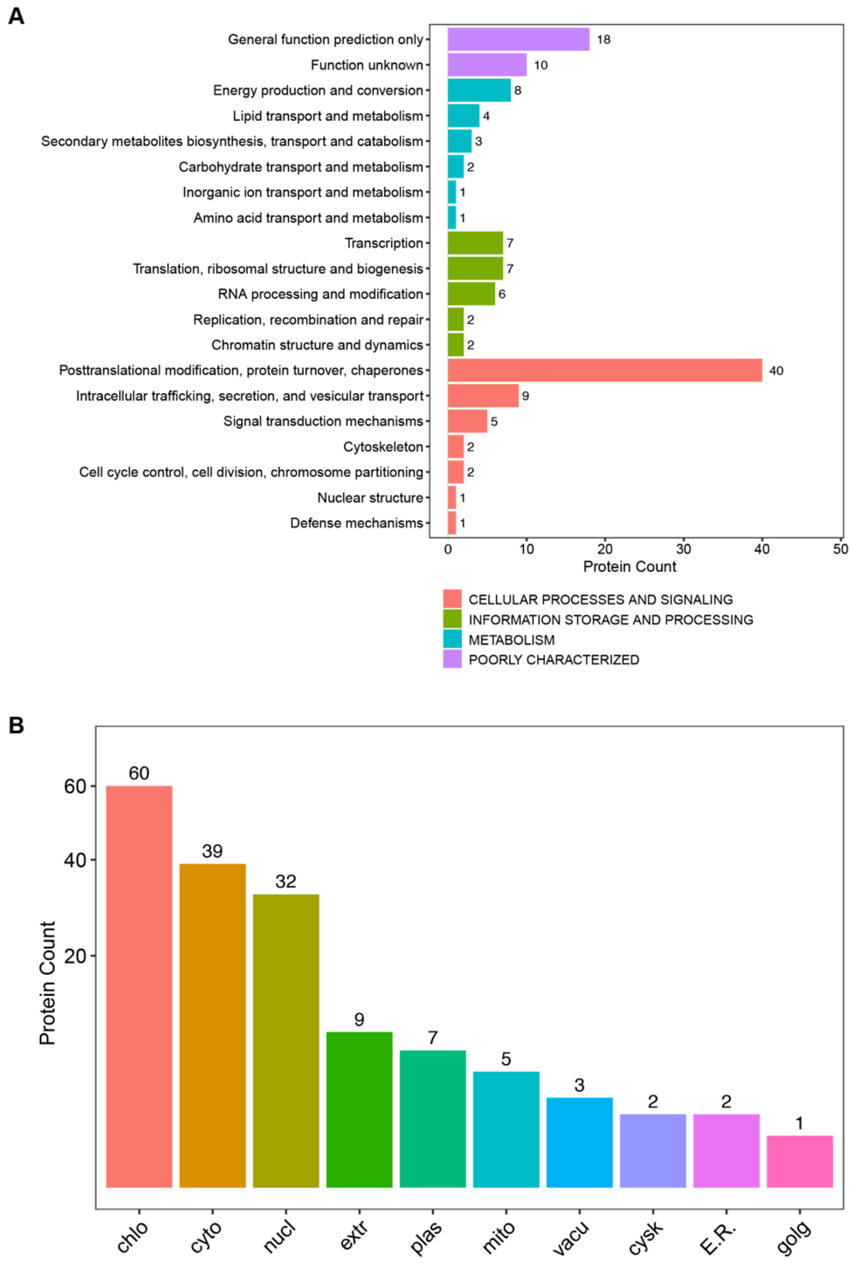

Supplementary Figure S3. KOG annotation and subcellular localization of DAPs in AC/NA. **(A)** Categories of KOG annotation. **(B)** Categories of subcellular localization. chlo, chloroplast; cyto, cytosol; nucl, nucleus; extr, extracellular; plas, plasma membrane; mito, mitochondria; vacu, vacuolar membrane; cysk, cytoskeleton; E.R., endoplasmic reticulum; golg, Golgi apparatus.

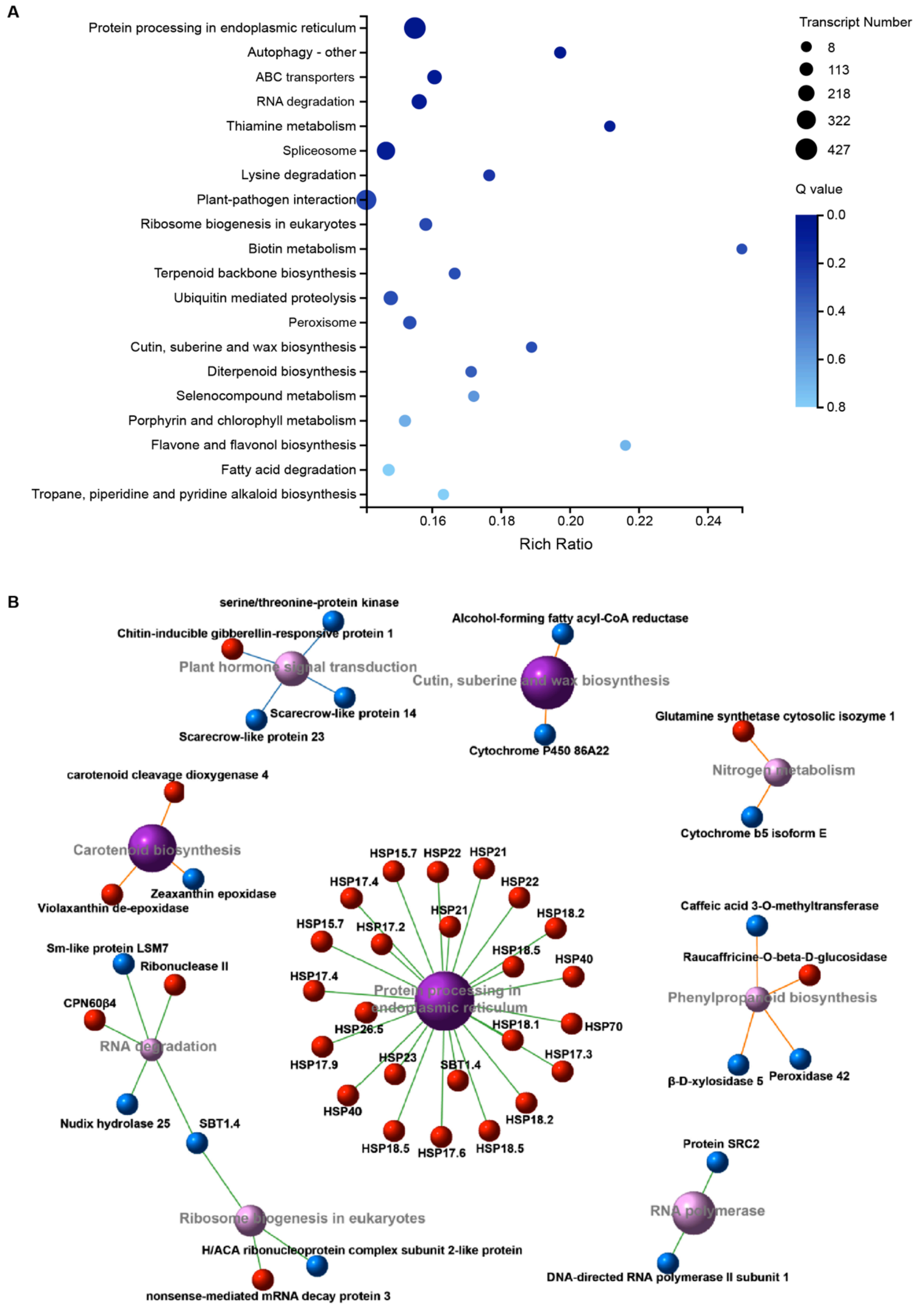

Supplementary Figure S4. KEGG pathway enrichment of DETs and DAPs in AR/NA. **(A)** KEGG pathway enrichment of DETs in AC/NA. Bubble size indicates transcript number and bubble color indicates Q value. **(B)** KEGG pathway enrichment of DAPs in comparison of AR/NA. Red and blue balls represent up- and downregulated proteins, respectively. Purple balls indicate top eight enriched

pathways, with dark color meaning significantly enriched and light color meaning enriched but not significantly, and larger areas indicate higher levels of enrichment. Different colors of line represent different classifications of pathway: red line indicates “cellular processes”, blue line indicates “environmental information processing”, green line indicates “genetic information processing” and orange line indicates “metabolism”. The detailed information of the DAPs was listed in Data S2.

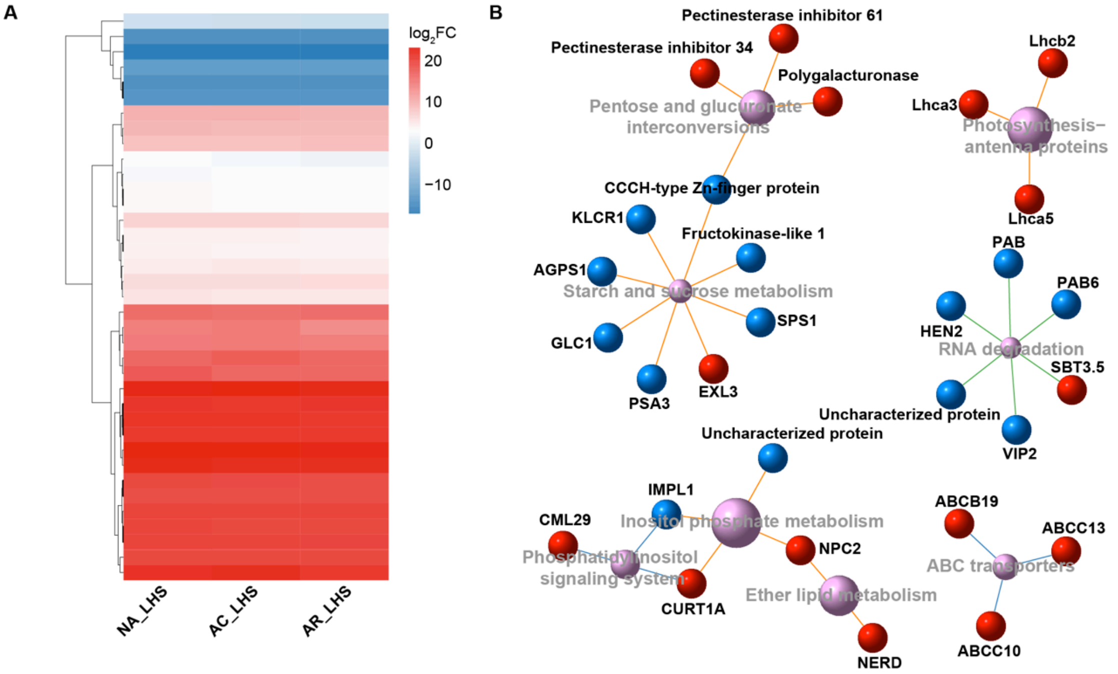

Supplementary Figure S5. Proteomic profiles after LHS. **(A)** Heat map of DAPs that highly accumulated in AC and AR. They show no significant difference among NA_LHS, AC_LHS and AR_LHS. The protein values are the averages from three biological replicates, normalized to the NA, and then log2 transformed. The detailed information of the DAPs was listed in Data S7. **(B)** KEGG pathway enrichment of AR_HS/NA_HS. Red and blue balls represent up- and downregulated proteins, respectively. Purple balls indicate top eight enriched pathways, with dark color meaning significantly enriched and light color meaning enriched but not significantly, and larger areas indicate higher levels of enrichment. Different colors of line represent different classifications of pathway: blue line indicates "environmental information processing", green line indicates "genetic information processing" and orange line indicates "metabolism".

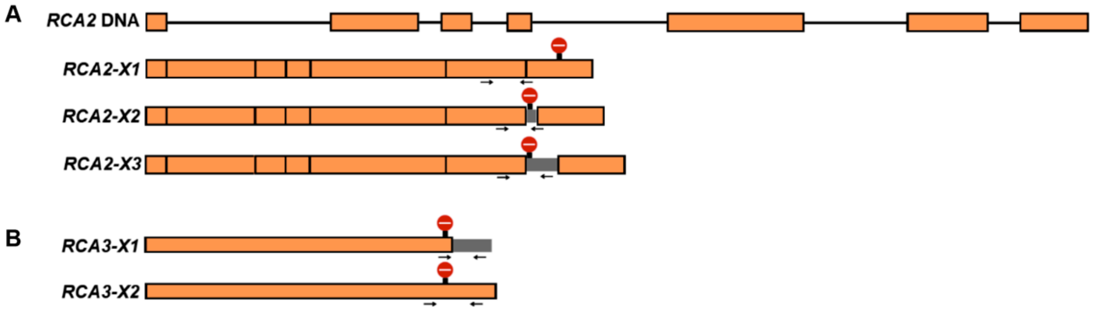

Supplementary Figure S6. Schematic diagram of **(A)** *RCA2* and **(B)** *RCA3* transcript structures. Orange module represents exon and line represents intron. Stop sign indicates position of termination codon. Gray module represents retained intron. Positions of specific primers for qRT-PCR were marked as arrows and primer sequences were listed in Table S1. For *RCA3* transcripts, that doesn't mean an exon because we haven't got the genomic DNA sequence, which may due to a very long genomic DNA sequence of *RCA3* like *Vitis vinifera*, whose *RCA3* has 51,401 nucleotides (NCBI Gene ID: LOC100263841).

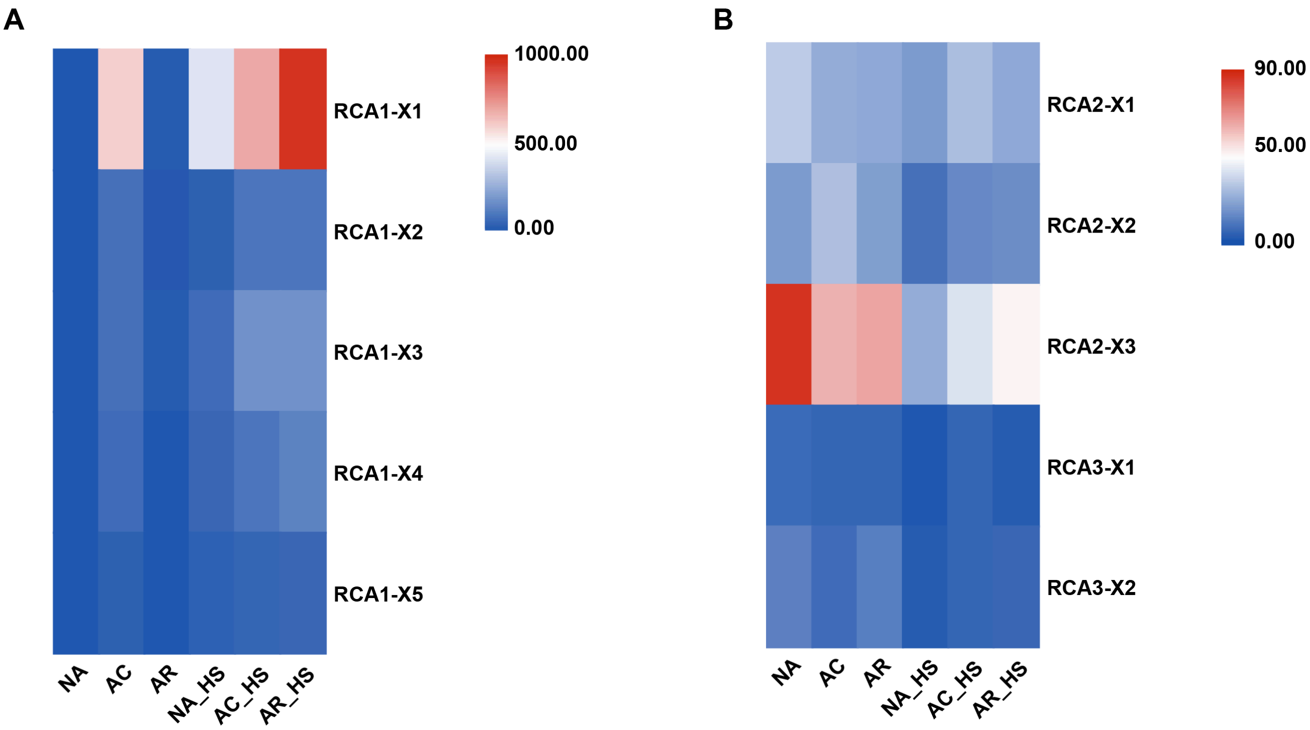

Supplementary Figure S7. FPKM values of alternative splicing transcripts of **(A)** *RCA1* and **(B)** *RCA2* and *RCA3*.

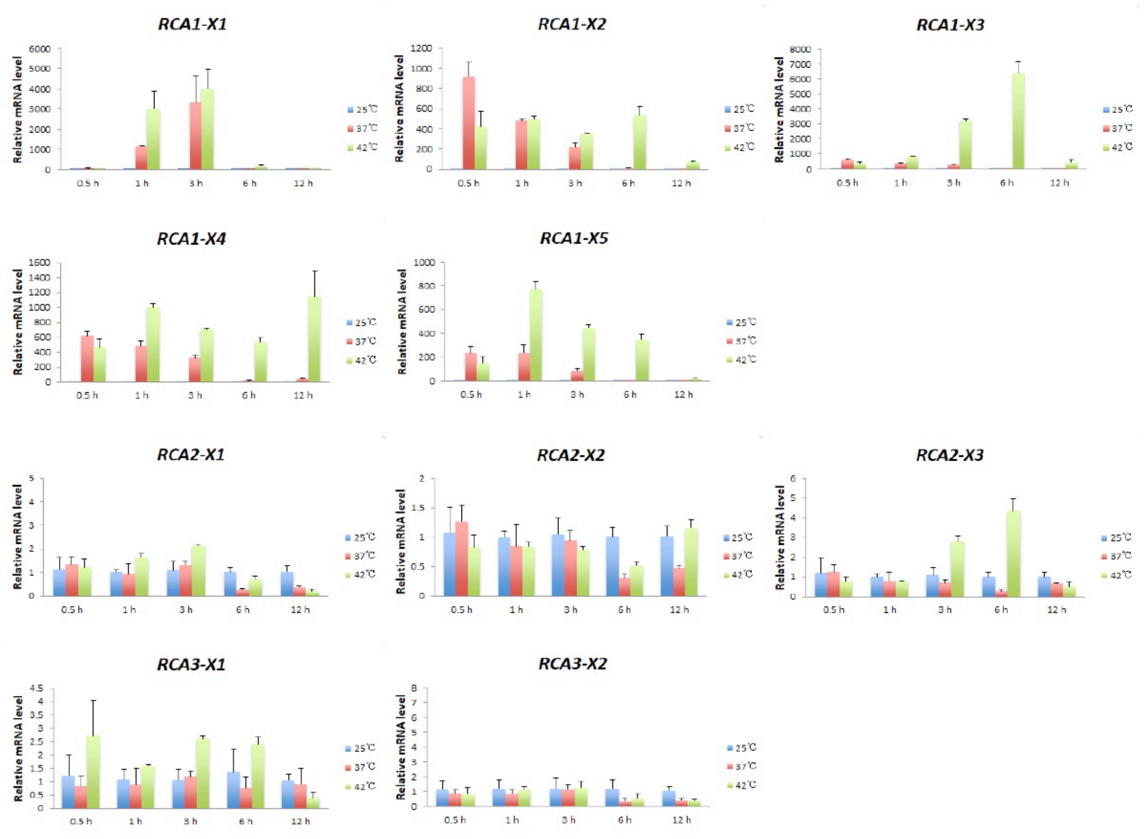

Supplementary Figure S8. qRT-PCR analysis of alternative-spliced *RCA* transcripts. For each time point, values of 37℃ and 42℃ were normalized to 25℃ to eliminate effects of time change. Data are shown as the relative mean values ± standard deviation (SD) of three biological replicates. Primers for qRT-PCR were listed in Table S1.
